# Supplementary material for: Perinatal Lead (Pb) Exposure and Cortical Neuron-Specific DNA Methylation in Male Mice
Source: Genes (Basel). 2019 Apr 4;10(4):274. doi: 10.3390/genes10040274 (PMC6523909; doi:10.3390/genes10040274)
Supplement: Supplementary file 1 [file genes-10-00274-s001.zip › Supplemental Table and Figures.docx]

**Supplement**

Perinatal lead (Pb) exposure and cortical neuron-specific DNA methylation in mice

| **Supplementary Table 1: Number of mice used in pooling scheme** | | | |
| --- | --- | --- | --- |
|  | **Pooled Sample 1** | **Pooled Sample 2** | **Pooled Sample 3** |
| **0 ppm** | 2 mice | 2 mice | 2 mice |
| **2.1 ppm** | 2 mice | 2 mice | 2 mice |
| **32 ppm** | 3 mice | 2 mice | 2 mice |

**Supplementary Figure 1 – Fluorescence-activated Cell Sorting (FACS) to separate cortical nuclei that are NeuN^+^ (neuronal origin) or NeuN^-^ (non-neuronal in origin).**


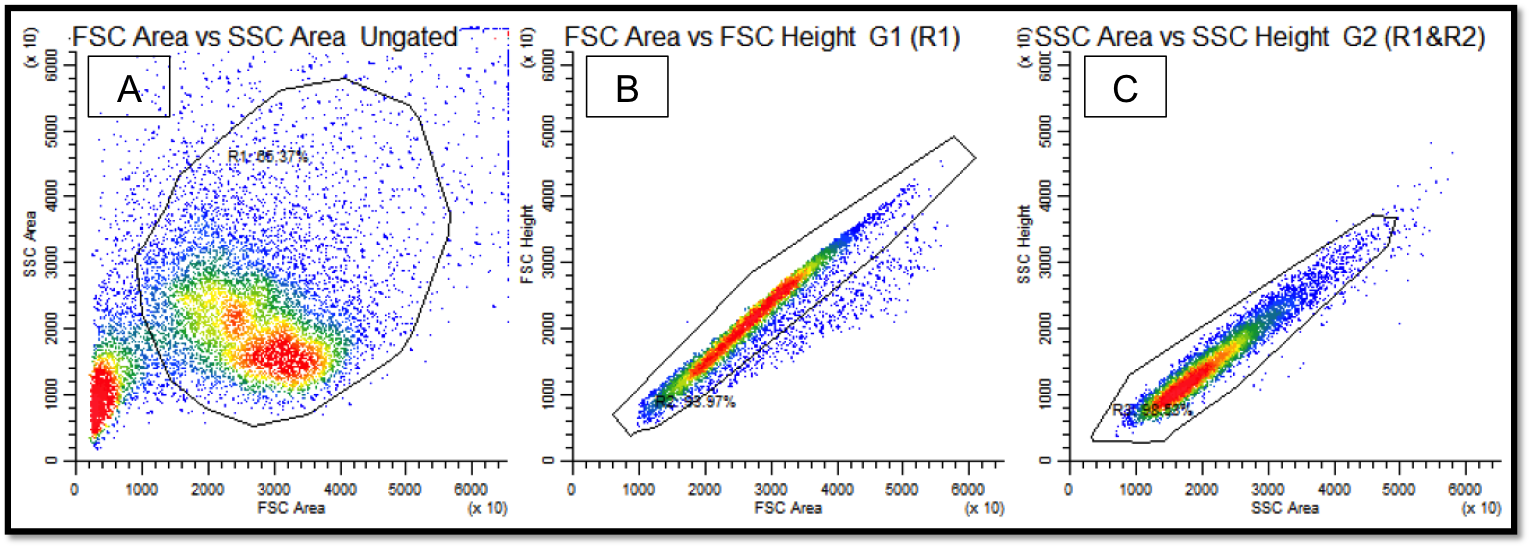


**A)** Plot of forward scatter-area vs side scatter-area to filter out debris from nuclei population (encircled area, Gate R1)

**B and C**) – Plots of forward scatter-area vs forward scatter-height **(B)** and side scatter-area vs side scatter-height **(C)** to remove doublets.

**Supplementary Figure 1 (cont) – Fluorescence-activated Cell Sorting**


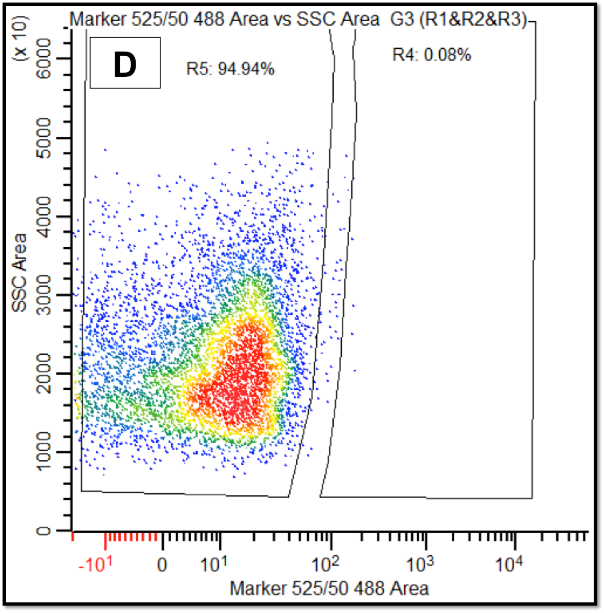

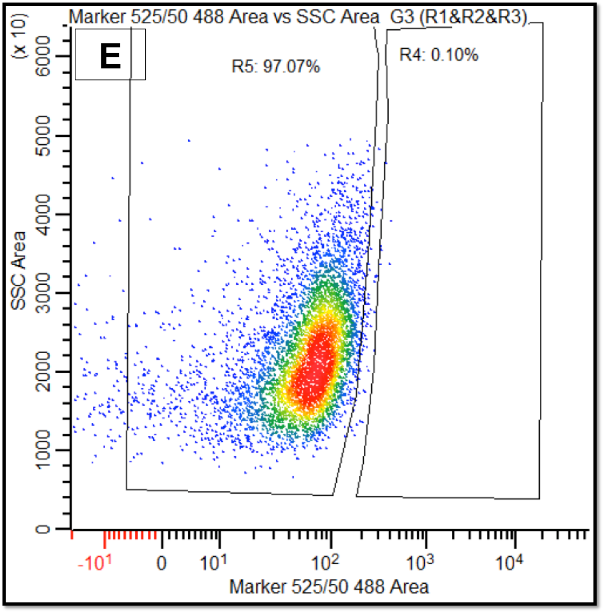

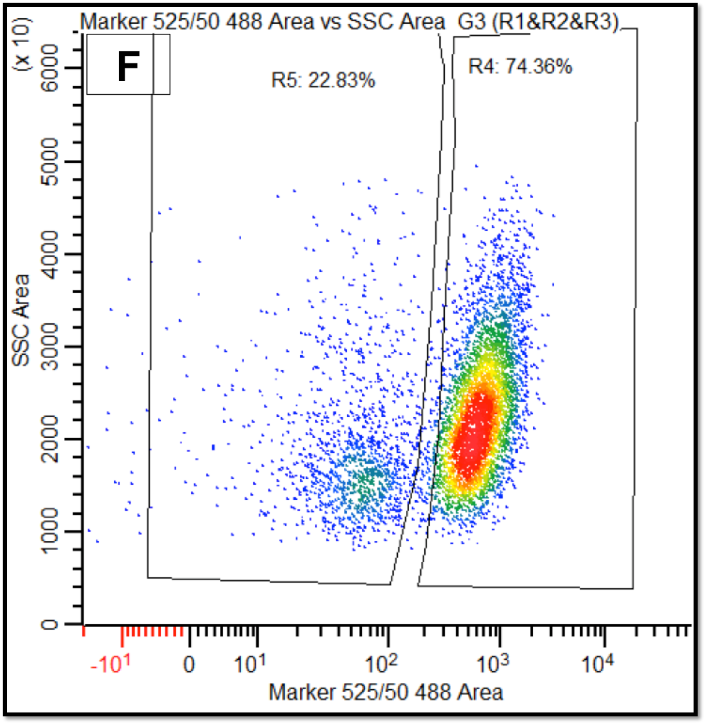


**D)** Unstained control. **E)** Saturation control, pre-incubated with untagged Anti-NeuN, to determine non-specific binding gate. **F)** Separation of AlexaFluor488-Anti-NeuN^+^ neuronal nuclei in Gate R4 vs non-neuronal Anti-NeuN^-^ nuclei in Gate R5.

**Supplementary Figure 2 – Fragmentation of NeuN(+) DNA via sonication**


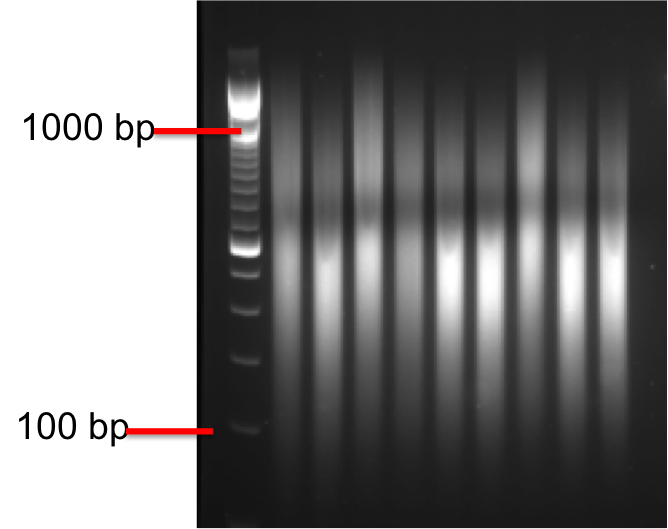
**A**


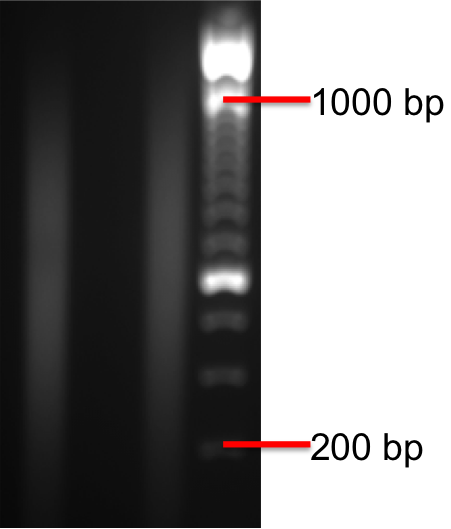


**B**

**A)** Pooled samples 1-9. Samples 3 and 7, corresponding to pool 3 for control group and pool 1 for high dose group, did not reach appropriate fragment size.

**B)** Pooled samples 3 and 7, corresponded to lanes 1 and 3, respectively. After re-sonication, the samples reached appropriate fragment size.
